# Dasatinib Inhibits Procoagulant and Clot Retracting Activities of Human Platelets

**DOI:** 10.3390/ijms20215430

**Published:** 2019-10-31

**Authors:** Ildikó Beke Debreceni, Gabriella Mezei, Péter Batár, Árpád Illés, János Kappelmayer

**Affiliations:** 1 Department of Laboratory Medicine, Faculty of Medicine, University of Debrecen, Kálmán Laki Doctoral School, 4032 Debrecen, Hungary; ideb@med.unideb.hu; 2 Division of Hematology, Department of Internal Medicine, Faculty of Medicine, University of Debrecen, 4032 Debrecen, Hungary; gmezei@med.unideb.hu (G.M.); pbatar@med.unideb.hu (P.B.); illes.arpad@med.unideb.hu (Á.I.)

**Keywords:** bleeding, dasatinib therapy, glycoprotein VI, platelet activation, thrombin formation

## Abstract

Tyrosine kinase inhibitors (TKI) such as the BCR-ABL inhibitor dasatinib and nilotinib are highly effective therapies for chronic myeloid leukemia (CML). However, several lines of evidence suggest that dasatinib can induce bleeding which may be due to impaired collagen-induced platelet adhesion, aggregation, and secretion. Sarcoma family kinases (SFK) play central role in the GPVI-induced signaling pathway. We aimed to investigate whether and how dasatinib can modulate SFK-mediated platelet procoagulant activity in a purified system and in dasatinib/nilotinib treated CML patients. In platelet rich plasmas of healthy volunteers, dasatinib dose-dependently reduced convulxin-induced phosphatidylserine exposure and attenuated thrombin formation. Similarly to these changes, integrin activation and clot retraction were also significantly inhibited by 100 nM dasatinib. Platelets isolated from dasatinib treated patients showed a significantly lower phosphatidylserine expression upon convulxin activation compared to premedication levels. In these samples, thrombin generation was significantly slower, and the quantity of formed thrombin was less compared to the trough sample. Western blot analyses showed decreased phosphorylation levels of the C-terminal tail and the activation loop of SFKs upon dasatinib administration. Taken together, these results suggest that dasatinib inhibits the formation of procoagulant platelets via the GPVI receptor by inhibiting phosphorylation of SFKs.

## 1. Introduction

Tyrosine kinase inhibitors (TKIs) are widely used drugs as a targeted strategy for cancer treatment with the aim of prolonging progression-free survival. Deregulated tyrosine kinase activity of the BCR-ABL oncoprotein is the biochemical hallmark of Philadelphia chromosome-positive (Ph+) hematological malignancies. Currently, several generations of BCR-ABL TKIs are in clinical use for treatment of these malignancies. Introduction of the second-generation TKIs, nilotinib and dasatinib as first-line treatment resulted in rapid and deep reduction of BCR/ABL1 allele transcripts, and this provided a possibility for long-term survival in CML [1,2]. In addition to CML, several other patient populations were identified that undoubtedly benefited from TKI treatment. Patients with Ph+ adult acute lymphoblastic leukemia (ALL) may also benefit from an alternate TKI therapy [3]. In the treatment of pediatric patients with Ph+ ALL the addition of TKIs to conventional chemotherapy has improved outcomes of patients [4]. It has long been known that second-generation TKIs may have side effects as nilotinib can potentiate a prothrombotic state [5] while dasatinib is known to cause platelet dysfunction e.g. impaired collagen-induced platelet adhesion and aggregation [6,7]. Although both drugs bind to the ATP binding site of the kinase domain of the BCR/ABL protein they have different off target inhibitory effect on several other tyrosine kinases. Dasatinib is a potent multikinase inhibitor, including c-KIT, EPHA2, platelet-derived growth factor receptor-β, and SFKs. Nilotinib is also a second generation TKI with a broad inhibitory spectrum of various tyrosine kinases (PDGFR, c-KIT, ARG, EPHB4), but it does not inhibit SFKs [8]. Previous in vitro and ex vivo studies with dasatinib have demonstrated a faulty platelet aggregation. On the contrary nilotinib has no effect on platelet aggregation, at all. Therefore, we used nilotinib as a negative control in our experiments.

Sarcoma family kinases (SFKs) are critical regulators of platelet signaling and activation. These kinases play a central role in mediating rapid response of platelets to vascular injury. They transmit activation signals from several various platelet receptors. There are numerous members of this group and among the SFKs Lyn, Fyn, and Src have been implicated in activation of the GPVI receptor and the integrin receptor signaling and are frequently studied proteins as they are present in both human and mouse platelets [9]. TKIs have a different off-target multikinase inhibitory effect. Comprehensive drug-protein interaction profiles were described [10] to predict the potential side effects of BCR-ABL TKIs. It was found that the most prominent dasatinib-targeted SFKs are Lyn, Fyn, and Src, kinases and their negative regulator C-terminal Src kinase (Csk), but nilotinib does not bind to these kinases. During vascular injury it is primarily the subendothelial collagen that activates platelets and result in subsequent platelet aggregation. A population of the collagen-adhered platelets responds by surface exposure of the procoagulant phosphatidylserine (PS). This surface expressed PS facilitates the binding of coagulation factors and by this promotes thrombin generation [11]. In addition, platelets participate in fibrin formation and regulate the process of clot retraction [12].

We intended to investigate whether dasatinib also affects the platelet procoagulant activity and thereby coagulation. For this reason, we examined the in vitro and ex vivo effects of dasatinib on platelet procoagulant response in dasatinib treated platelets of healthy volunteers and in samples derived from CML patients on dasatinib therapy. We found that at therapeutic concentration dasatainib, but not nilotinib, has a strong inhibitory effect on platelet procoagulant activity and on clot retraction both in non activated as well as in convulxin activated platelets.

## 2. Results

### 2.1. Dasatinib Suppresses Convulxin-Induced PS Exposure and Attenuates Thrombin Generation in Vitro

**Figure 1:**
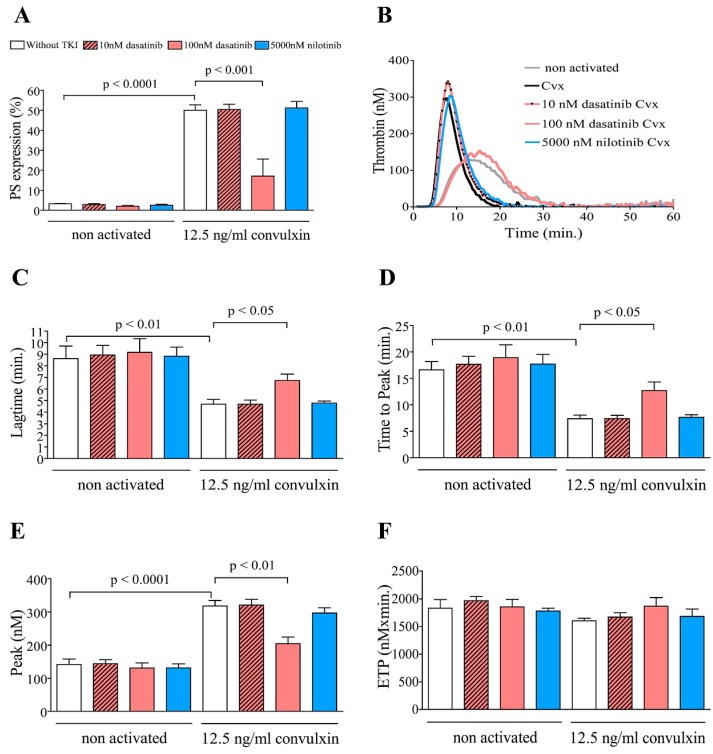
Dasatinib inhibits convulxin-induced procoagulant phosphatidylserine (PS) exposure and thrombin generation in vitro. Platelet rich plasma (PRP) from healthy donors were incubated with 10 or 100 nM dasatinib, 5000 nM nilotinib or vehicle (0.2% DMSO) for 10 min. Platelets, pretreated with vehicle (without Tyrosine kinase inhibitors (TKI)) or TKI, subsequently they were stimulated with 12.5 ng/mL convulxin for 15 min. Exposure of PS was determined by FITC-labeled annexin V (**A**). The histogram shows percentages of platelets binding FITC-annexin V. Representative thrombin generation curves of TKI pretreated and convulxin stimulated PRPs (**B**). Lagtime (**C**), time to peak (**D**), peak thrombin (**E**), and Endogenous Thrombin Potential (ETP) (**F**) parameters were evaluated. The results are the mean ± SEM of five different experiments.

Platelets play a central role in the primary hemostasis and are also actively involved in cell-based thrombin generation. PS on platelets potentiates thrombin generation by harboring the coagulation factors. We first investigated the effect of dasatinib and nilotinib on the PS expression of resting and convulxin activated platelets. Pretreatment of platelets with 100 nM dasatinib caused significant reduction of PS expression even in non-activated samples. The GPVI agonist convulxin caused a definitely higher PS expression compared to non-activated platelets, which was abolished by 100 nM dasatinib pretreatment. Low concentration of dasatinib (10 nM) and nilotinib did not affect PS expression neither in non activated nor in convulxin activated samples (Figure 1A). In the same experiments, the modulatory effect of dasatinib on the thrombin generation was investigated. The thrombin generation test (TGT) can be executed both in platelet poor plasma (PPP) and in platelet rich plasma (PRP). In case of PPP thrombin generation is elicited by the simultaneous addition of tissue factor (TF) and phospholipids (PL) while in case of PRP the activator is only TF and the PL is provided by the activated platelet surface lipids. TGT is a more sophisticated test than the conventional PT and APTT as it monitors the whole process of thrombin formation while for the fibrin formation in the conventional clotting time assays roughly 5% of the total thrombin is sufficient. The Thrombinoscope software was used to monitor the rise and fall of thrombin formation in clotting PRP and these changes were illustrated by the thrombin generation curve (Thrombogram). Thrombograms were characterized by the following parameters calculated and presented by the Thrombinoscope software: (i) Lagtime, the moment at which thrombin generation starts (minutes); (ii) Time to Peak, the time until the Peak Thrombin (minutes); (iii) Peak Thrombin, the highest thrombin concentration (nM); (iv) Endogenous Thrombin Potential (ETP), the area under the curve (nM × min).

Representative thrombin generation curves demonstrate the effect of convulxin and dasatinib or nilotinib on thrombin formation by platelets (Figure 1B). Thrombin formation was faster, and the lagtime and time to peak values were significantly shorter in convulxin activated samples compared to the non activated samples (Figure 1C,D). Activation by convulxin increased the peak thrombin but did not affect the ETP values. High concentration of dasatinib (100 nM) significantly delayed thrombin generation in the convulxin activated samples but did not affect the non activated samples (Figure 1C,D). In addition, 100 nM dasatinib also significantly attenuated the peak thrombin but had no effect on the ETP (Figure 1E,F) in the convulxin activated sample.

### 2.2. Dasatinib Inhibits the Activation of Integrin αIIbβ3 and Clot Retraction

**Figure 2:**
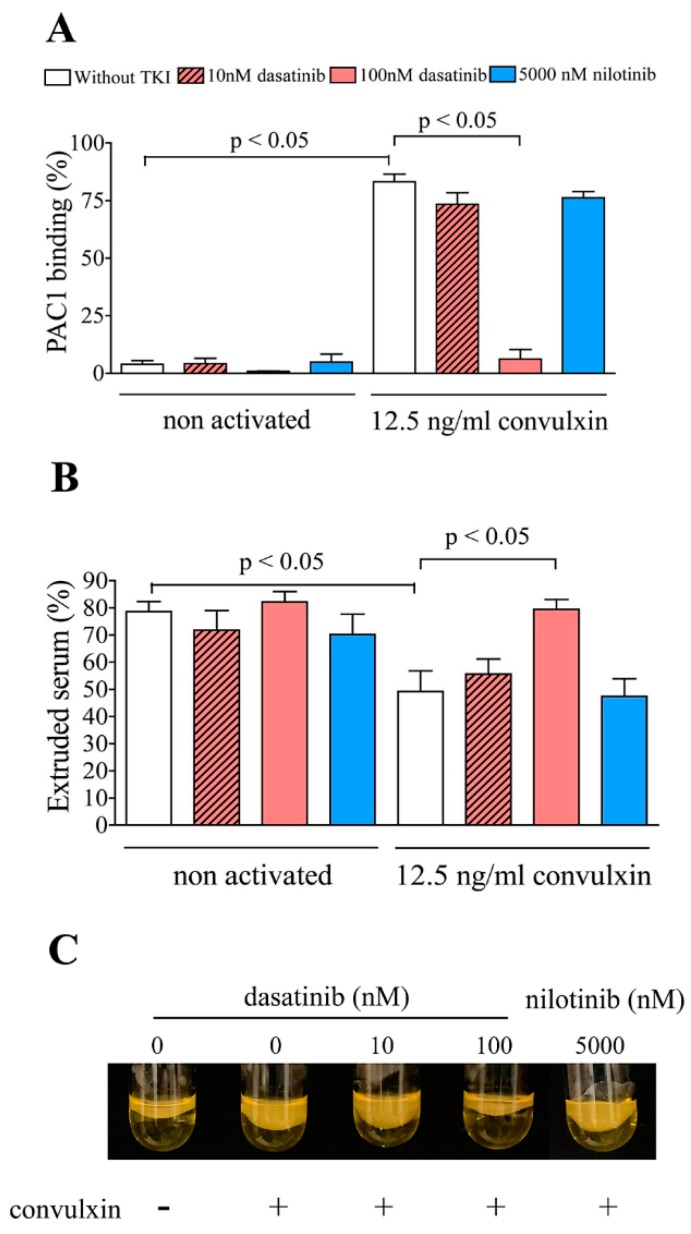
Inhibitory effect of dasatinib on convulxin-induced integrin αIIbβ3 activation and clot retraction. Platelets in control PRPs were pretreated with dasatinib/nilotinib or vehicle (without TKI) and subsequently were stimulated with convulxin. The active conformation of the integrin αIIbβ3 was detected by FITC-labeled PAC1 monoclonal antibody and analyzed by flow cytometry. The percentages of platelets binding FITC-PAC1 are shown (**A**). Clot retraction of platelets was elicited by high calcium concentration in TKI pretreated and convulxin-activated PRPs (**B**). Results were expressed as extruded percent of the original volume. The results are the mean ± SEM of 5 different experiments. Panel **C** shows a photo series to present the clot retraction phenomenon.

Flow cytometry was used to determine the effect of dasatinib on integrin αIIbβ3 activation by PAC1 binding. Dasatinib at 100 nM significantly reduced the level of activated integrin in non activated sample and strongly inhibited the integrin activation that was elicited by the GPVI receptor agonist, convulxin (Figure 2A). Clot retraction was assayed for 60 min. At the end of the incubation period, the non activated sample displayed intense clot retraction. In samples, where platelets were activated by convulxin, the clot contraction was less intense. However, 100 nM dasatinib pretreatment significantly increased the volume of the extruded serum in the convulxin activated sample (Figure 2B,C). Nilotinib pretreatment had no effect on integrin activation or clot retraction.

### 2.3. Dasatinib but Not Nilotinib Treatment of CML Patients Considerably Alters PS Exposure and Thrombin Generation

**Figure 3:**
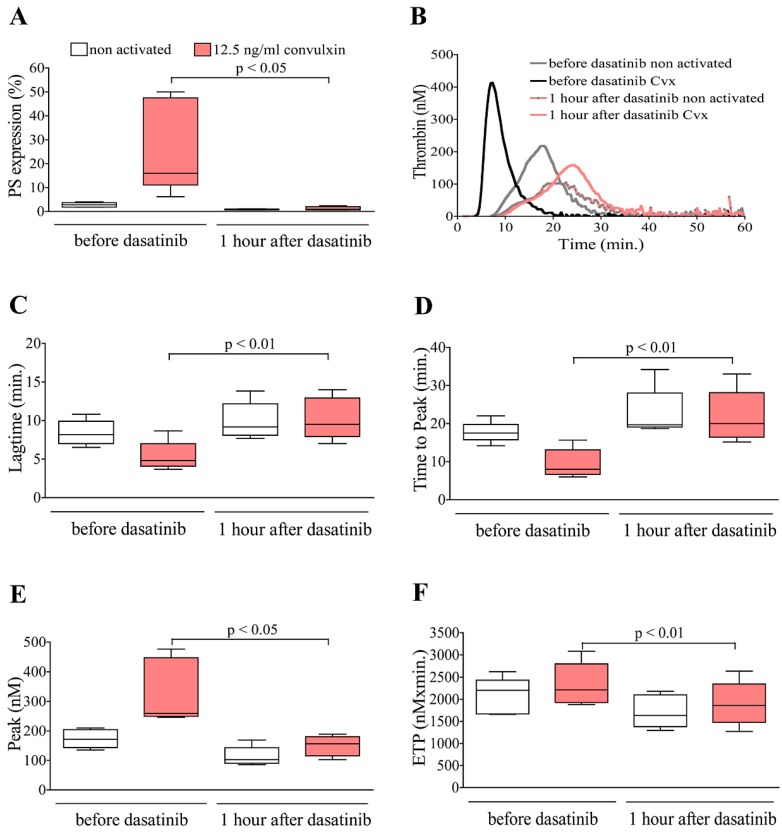
Treatment of chronic myeloid leukemia (CML) patients with dasatinib suppresses PS exposure and generation of thrombin in PRP. Blood was collected from dasatinib treated CML patients (*n* = 5) before and at 1 h after witnessed drug administration. Platelets of patients were stimulated with 12.5 ng/mL convulxin for 15 min in PRP. PS exposure (**A**) and thrombin generation were investigated. Panel (**B**) shows representative overlay thrombin generation curves. Time parameters of thrombin generation, lagtime (**C**); time to peak (**D**); and quantity of generated thrombin, peak (**E**) and ETP (**F**), were evaluated.

**Figure 4:**
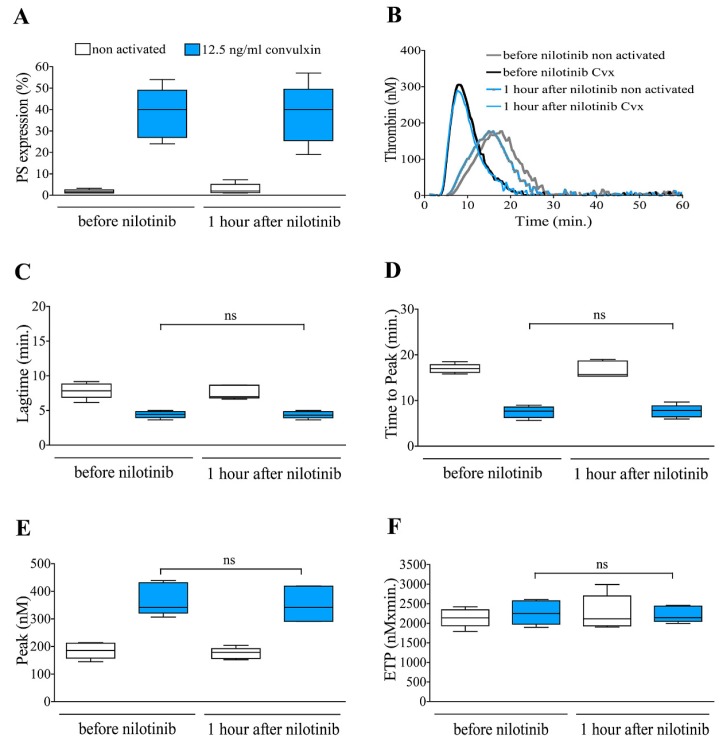
Nilotinib treatment does not affect PS expression and thrombin generation in PRP of CML patients. Samples were from nilotinib treated CML patients (*n* = 5) before and at 1 h after witnessed drug administration. Platelets of patients were stimulated with convulxin for 15 min in PRP. PS exposure (**A**) and thrombin generation were investigated. Panel (**B**) shows representative overlay thrombin generation curves. Time parameters of thrombin generation, lagtime (**C**); time to peak (**D**); and quantity of generated thrombin, peak (**E**) and ETP (**F**), were evaluated.

Peripheral blood samples of TKI treated CML patients were drawn immediately before and at 1 h after witnessed dasatinib/nilotinib administration. PRPs were separated by centrifugation, and a part of the samples were activated by convulxin. Similarly to the in vitro experiments, the procoagulant activity of platelets was examined by detecting PS expression and measuring thrombin generation. The PS expression was completely suppressed on the platelet surface at 1 h after dasatinib treatment both in non activated and convulxin activated samples (Figure 3A). Nilotinib treatment had no inhibitory effect on PS expression neither in non activated nor in convulxin activated samples (Figure 4A). Effect of dasatinib/nilotinib treatment on thrombin generation was shown by representative thrombograms (Figure 3B and Figure 4B). The kinetics of thrombin generation, (lagtime and time to peak) as well as the quantity of generated thrombin (thrombin peak and ETP) were studied. The time parameters of thrombin formation were shorter, and the amount of generated thrombin was greater upon convulxin activation in samples before dasatinib treatment. At 1 h after dasatinib administration the time for thrombin generation was prolonged (Figure 3C,D) and the quantity of the formed thrombin—peak and ETP values—were significantly reduced (Figure 3E,F) compared to the premedication samples in both non activated and convulxin activated samples. In premedication samples of the nilotinib treated group, convulxin caused similar changes in thrombin generation than in premedication sample of dasatinib treated group, the time parameters were shortened, and the quantity of generated thrombin was increased. Unlike in case of dasatinib, in the nilotinib treated group, we could not observe any difference in thrombin generation between the premedication and postmedication samples (Figure 4C–F).

### 2.4. Integrin Activation and Clot Retraction were Considerably Affected in PRPs of Dasatinib Treated CML Patients

**Figure 5:**
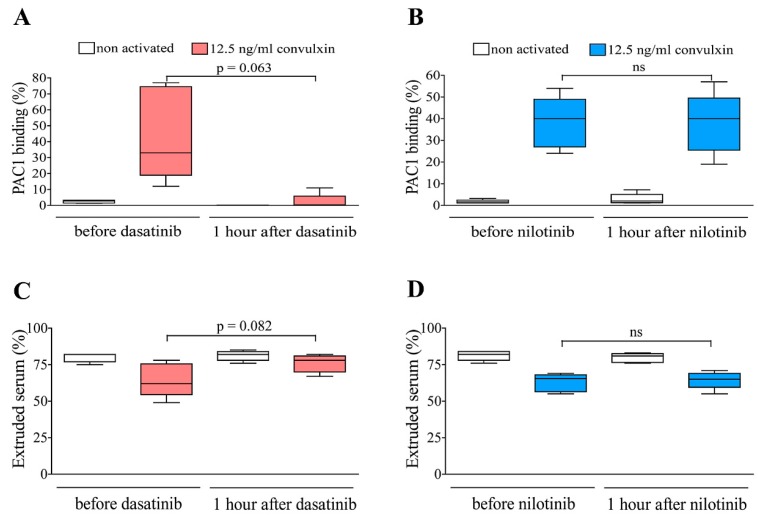
Integrin αIIbβ3 activation and clot retraction in PRPs of TKI treated CML patients. Blood was collected from dasatinib (*n* = 5) or nilotinib (*n* = 5) treated CML patients before and at 1 h after witnessed drug administration. Platelets of patients were stimulated with convulxin in PRP. The percent of active conformation of integrin αIIbβ3 is shown by FITC-PAC1 binding in the cases of dasatinib or nilotinib treated patients respectively (Panels **A** and **B**). Panels **C** and **D** show results of clot retraction in PRPs of dasatinib or nilotinib treated patients.

Low level of activated integrin αIIbβ3 was observed on the platelets of dasatinib treated group in the premedication samples, and it was further decreased at 1 h after dasatinib medication. The amount of activated integrin was increased in the premedication sample of both dasatinib and nilotinib treated groups upon convulxin stimulation. However, dasatinib treatment considerably inhibited convulxin-induced integrin activation, whereas nilotinib did not (Figure 5A,B). It could be observed that clot retraction was intense and definitely similar in premedication and postmedication samples of dasatinib/nilotinib treated patients (Figure 5C,D). At the same time, the clot retraction became less intense by convulxin stimulation in premedication samples of the dasatinib or nilotinib treated groups. It could be observed that dasatinib treatment non significantly attenuated the effect of convulxin that is exemplified by a considerably increased volume of the extruded serum (Figure 5C).

### 2.5. Dasatinib Inhibits Phosphorylation of Regulatory Sites of SFKs

**Figure 6:**
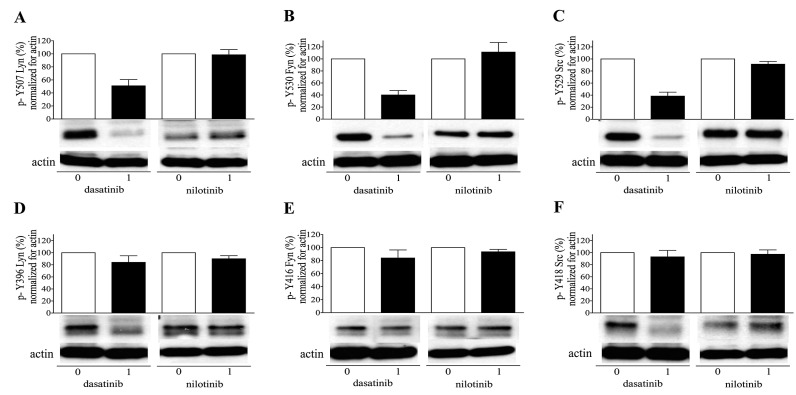
Dasatinib inhibits the phosphorylation of C-terminal tail and activation loop of Sarcoma family kinases (SFK) in CML patients. Lysates of platelets from dasatinib (*n* = 3) or nilotinib (*n* = 3) treated patients were examined with the indicated antibodies against the inhibitory (**A**–**C**) and the full activatory (**D**–**F**) phosphorylation of Lyn, Fyn, and Src kinases. The quantity of phosphorylated SFKs in platelet lysates before drug administration (0) was regarded as 100 %. Columns show the mean and SEM.

**Figure 7:**
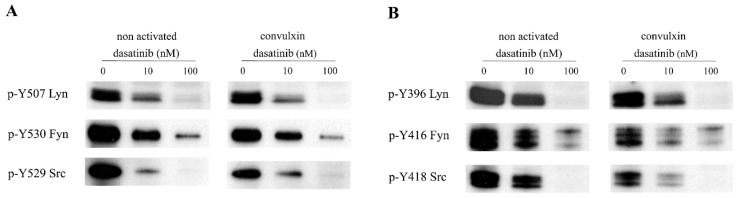
Phosphorylation status of SFK after dasatinib treatment upon convulxin activation. Gel filtrated human platelets from a healthy donor were incubated with 10 or 100 nM dasatinib or vehicle (0.2% DMSO) for 10 min. After incubation, platelets were left non activated or were activated with 12.5 ng/mL convulxin for 15 min. Platelet lysates were immunoblotted with phosphospecific antibodies against the inhibitory (**A**) and the full activatory (**B**) phosphorylation of Lyn, Fyn, and Src kinases.

The decreased platelet response to the GPVI agonist convulxin suggested that dasatinib may influence platelet signaling via the inhibition of SFKs. First, the platelet lysates of dasatinib/nilotinib treated CML patients were examined using western blot. We observed that at 1 h after dasatinib treatment, the inhibitory phosphorylations of Fyn, Lyn, and Src kinases were remarkably reduced (Figure 6A–C) while the activation loop phosphorylations were only slightly suppressed or not at all (Figure 6D,F) compared to the premedication samples. Nilotinib treatment did not cause marked change in the phosphorylation level of the regulatory sites. In order to understand how dasatinib can affect platelet activation, we examined tyrosine phosphorylation of both regulatory sites of SFKs in a series of in vitro experiments. Control platelets were treated by dasatinib at 0, 10, and 100 nM final concentration, and these TKI pretreated platelets were activated by convulxin. In case platelets were not pretreated with dasatinib (0 nM) but were activated by convulxin for 15 min, a decreased phosphorylation level was observed in both the C-terminal tail and the activation loop of SFKs. In line with the previous data, convulxin could result in an attenuated effect in the presence of 10 nM dasatinib but 100 nM dasatinib abolished this effect (Figure 7).

## 3. Discussion

The two investigated drugs in this study, although both belong to the second-generation of the TKIs, exert their off-target effect differently. Dasatinib is a Type I while Nilotinib is a Type II inhibitor. Type I inhibitors bind at the ATP-binding pocket, which is highly conserved across the human kinome, and to achieve greater selectivity than ATP, Type I inhibitors typically not only occupy the space where the ATP adenine group binds but also extends into different proximal regions. Type II inhibitors bind not only to the ATP adenine group area but also into the allosteric pocket with the benzamide substituent. Since the ATP-binding site on diverse kinases in the human body is structurally conserved, it is expected that these compounds may have unintended inhibitory actions at nontarget kinases. Indeed, several lines of evidence suggest that multi-target TKIs can have a wide range of side effects. Nilotinib activates the endothelium and platelets in vitro and in vivo and moreover potentiates platelet adhesion and thrombus formation [5], thus may elicit prothrombotic or atherogenic effects [13,14]. Contrary to nilotinib, dasatinib was shown to elicit hemorrhagic adverse events in CML patients [15,16]. In vitro and ex vivo studies demonstrated its effect on primary hemostasis. Dasatinib reversibly inhibits collagen-induced platelet adhesion, activation, and aggregation, and these are mediated by inhibition of the Src kinase [6]. Similarly to these findings, in our previous study we could observe an inhibitory effect of dasatinib on collagen-induced platelet aggregation and ATP secretion in in vitro experiments and ex vivo samples as well [17].

Platelets, upon activation by various agonists, are known to form populations with different surface properties. These populations of activated platelets also have different functions depending on their activation state and surface properties. One population of stimulated platelets shows high level of activated integrin αIIbβ3 to which fibrinogen can bind and this results in platelet–platelet and platelet–leukocyte aggregation and clot-retraction. Another population exposes surface PS, and these cells are described as procoagulant platelets. The formation of PS-exposing platelets is linked to a prolonged, high cytosolic Ca2+ level that is required for swelling and phospholipid scrambling. In addition, these platelets are characterized by a calpain-mediated inactivation of the αIIbβ3 integrin and thus can not participate in clot retraction. Thus, there are major differences between aggregatory and procoagulant platelets.

Procoagulant platelets contribute to fibrin formation, which is initially linked to PS exposure and secondarily depend on integrin αIIbβ3 activation and transglutaminase-dependent fibrin cross-linking. Fibrin is preferentially localized near the sites of tissue factor and on procoagulant platelets. The procoagulant platelets are deposited on the thrombus surface during the contraction process that can be crucial for the spatial control of thrombin generation and fibrin formation [18]. Coated-platelets are a subpopulation of thrombocytes formed after stimulation with collagen plus thrombin also express PS and possess procoagulant properties [11,19]. In platelets, signaling via the GPVI receptor is a major pathway for the formation of procoagulant platelets [20].

In the present study, we demonstrate for the first time the effects of second-generation TKIs on the procoagulant activity of platelets. In our in vitro experiments, we used the clinically relevant concentrations of TKIs [21]. The results of the in vitro experiments clearly showed that at the higher end of the therapeutic range, dasatinib markedly reduced the convulxin-induced activation response of isolated platelets, including PS exposure, thrombin formation, integrin activation and subsequent clot retraction. The inhibitory effect on PS exposure and PAC1 expression was also evident in non activated platelets. Furthermore, in the ex vivo study carried out in CML patients at 1 h after dasatinib ingestion, the inhibitory effect of dasatinib on procoagulant properties of platelets was also already observed in both activated and non-activated samples.

When platelets were activated via the GPVI receptor after dasatinib pretreatment, the effect of convulxin was completely abolished in the PS dependent tests, and integrin activation and the clot-retraction were also attenuated. Nilotinib had no effect on the procoagulant activity of platelets neither in the in vitro nor in the ex vivo experiments. This is in line with results of our previous work where we found that therapeutic concentration of dasatinib reduced the coated-platelet generation, and nilotinib had no effect [17]. The inhibitory effect of dasatinib is not limited to the above activation markers; it also abolishes the alpha granule excretion in platelets that can be monitored by surface P-selectin expression (Figure S1).

Dasatinib can target SFKs, namely Lyn, Fyn, and Src kinases that are all critical regulators of platelet signaling and activation. The activity of SFK is regulated through the phosphorylation of conserved tyrosine residues in the C-terminal tail that inhibits SFK activity and in the activation loop that maximally active SFK.

Therefore, we studied the phoshorylation status of Lyn, Fyn, and Src at both regulatory sites in platelets of dasatinib or nilotinib treated CML patients. From our results, we concluded that dasatinib but not nilotinib inhibits both the maximally active and inactive form of these kinases. This observation is in accordance with results of the activity-based kinase profiling where dasatinib was found to inhibit nearly twice as much kinases than nilotinib [22]. The striking difference between the effect of dasatinib and nilotinib on platelet procoagulant activity suggests that the major effect of dasatinib may be mediated by SFK inhibition.

However, from these data, it cannot be concluded that dasatinib directly exerts its inhibitory effect on platelet procoagulant activity through inhibition of SFKs. Therefore, we examined the phosphorylation status of SFKs in dasatinib pretreated and convulxin activated platelets. In agreement with previous reports, we found a decreased phosphorylation level in both the C-terminal tail and the activation loop of SFKs [23,24] in platelets that were not pretreated with TKI but were activated by convulxin. In addition, we observed that already at the low end of the therapeutic concentration dasatinib (10 nM) considerably inhibited both form of SFKs in non activated platelets while at the high end (100 nM) of the dasatinib therapeutic range SFKs were totally inhibited. In line with previous changes, when platelets were pretreated with 10 nM dasatinib before activation, convulxin was able to exert its effect on both forms of SFKs, but 100 nM dasatinib completely abolished this effect. These effects were the same as we observed for PS expression in the in vitro experiments.

It has long been observed that dasatinib treatment may lead to bleeding symptoms in CML, which may be due to various reasons. On the one hand, dasatinib treatment may be associated with mild thrombocytopenia and an increased risk of bleeding [25]. We could not observe any clinical bleeding symptoms in our dasatinib treated patients and only 1 out of 5 was thrombocytopenic that further decreased by 29% upon dasatinib intake. On the other hand, dasatinib may cause platelet dysfunction but no association was found between bleeding symptoms and the impaired platelet function, and it was concluded that the occurrence of bleeding cannot be predicted by in vitro platelet aggregation tests [26]. In addition, dasatinib may exert an effect on other elements in the circulation that can considerably modify the side effects in dasatinib-treated CML patients. Dasatinib triggers a transient increase in vascular leakage that probably contributes to adverse effects such as bleeding diathesis. The side effect of dasatinib on the induction of eryptosis in human erythrocytes should not be neglected. Eryptosis is characterized by cell shrinkage, PS externalization and loss of membrane integrity. Eryptosis might promote blood coagulation through PS externalization. The effect of dasatinib on circulation is complex, and the clinical outcome of bleeding might depend on which effect is ore pronounced, e.g, inhibition or promotion of PS expression on different types of cells [27,28].

In summary, our work demonstrates a novel off-target effect of dasatinib on platelet function and describes the mechanisms that may lead to the observed phenomena. Our results are in accordance with conclusions of previous studies since although significant platelet function impairment was also evident in patients, this did not translate into any clinically significant bleeding. Dasatinib treatment results in a strong inhibition of GPVI receptor agonist-induced platelet procoagulant activity in vitro and in CML patients, and this effect may contribute to hemorrhagic consequences of dasatinib treated patients with endothelial disruption or damage when GPVI activating agonists like subendothelial collagen is exposed.

## 4. Materials and Methods

### 4.1. Materials

Dasatinib and nilotinib for in vitro experiments were from Cayman Chemical (Ann Arbor, MI, USA). The following directly conjugated monoclonal antibodies were purchased from Becton Dickinson (San Jose, CA, USA): annexin V-FITC, CD41a-PECy5, CD42a-FITC, CD62P-PE, PAC1-FITC. CD41-PE was from DAKO (Glostrup, Denmark). Dimethylsulfoxide (DMSO), Sepharose CL-2B, anti-actin and biotin conjugated anti-rabbit IgG were from Sigma-Aldrich (St. Louis, MO, USA) and convulxin were from Pentapharm (Basel, Switzerland). For western blot we used the following polyclonal antibodies: p-Fyn Y530, p-Lyn Y396, p-Src Y529, and p-Src Y418 (Thermo Fischer Scientific, Rockford, IL, USA); p-Fyn Y416 and p-Lyn Y507 Biorbyt (San Francisco, CA, USA); and Cell Signaling Technology (Leiden, The Netherlands), respectively. Avidin-biotin complex kit was from Vector Laboratories (Burlingame, CA, USA), and ECL reagent was used from Millipore (Billerica, MA, USA).

### 4.2. Blood Drawing from Healthy Volunteers and Patients

Peripheral blood samples were drawn from five healthy volunteers into tubes containing 0.105 M sodium citrate.

Healthy volunteers were recruited from the staff of the Department of Laboratory Medicine. Patients with chronic phase CML on dasatinib (*n* = 5) or nilotinib (*n* = 5) therapy were included in the ex vivo study. All dasatinib treated patients were taking 100 mg QD, and nilotinib treated patients were on 400 mg BID. Patient’s blood was drawn immediately before and 1 h after witnessed drug administration. Subjects were recruited from the Hematology Outpatient Clinic at the Institute of Internal Medicine University of Debrecen. Antiplatelet therapy, if any, was suspended 7 days prior to examination. At the time the samples were taken, all CML patients were on continuous dasatinib/nilotinib treatment for at least 4 weeks. Informed consent was obtained from all participants (CML patients and healthy volunteers) in accordance with the local institution review board guidelines. Ethical agreements were provided by the local ethical committee of the University of Debrecen (identification code: RKEB/IKEB 4875-2017, approval date: September 25, 2017).

### 4.3. Preparation of Platelet Rich Plasma (PRP) and Design of in Vitro and ex Vivo Study

PRP was prepared from whole blood by centrifugation at 170× *g* for 15 min at room temperature (RT). Platelet count of PRP was adjusted to 250 G/L by adding platelet poor plasma (PPP). PPP was obtained by centrifugation of the citrated blood sample at 1500× *g* for 15 min at RT.

PRPs from healthy volunteers were used for in vitro experiments of dasatinib/nilotinib effects; the following PRP samples were prepared: (i) non activated, (ii) dasatinib or nilotinib pretreated non activated, (iii) convulxin activated, and (iv) dasatinib or nilotinib pretreated and convulxin activated. For pretreatment dasatinib was used at 10 and 100 nM while nilotinib was used at 5000 nM final concentration for 10 min at 37 °C. Pretreated platelets were activated by GPVI agonist convulxin at 12.5 ng/mL final concentration for 15 min at 37 °C without stirring.

In the ex vivo study PRPs of dasatinib or nilotinib, treated CML patients were activated with convulxin at 12.5 ng/mL final concentration for 15 min at 37 °C without stirring thus, (i) non activated and (ii) convulxin activated samples were created.

### 4.4. Flow Cytometric Assays

Platelet PS expression was determined by annexin V binding to platelet surface using FITC-conjugated annexin V and for platelet identification PE-conjugated CD41 monoclonal antibody was used. Five microliter of PRP was stained with 5 µL of annexin V-FITC and 5 µL of CD41-PE in 35 µL annexin V binding buffer (it provides calcium for binding of annexin V), and the mixture was incubated for 15 min at RT in the dark. Active conformation of the integrin αIIbβ3 was determined by binding of FITC-conjugated PAC1 and PECy5-conjugated CD41a antibodies.

Samples were diluted to 550 µL with buffer and measured immediately after staining by an FC500 flow cytometer, and results were analyzed with the Kaluza software (Beckman Coulter, Brea, CA, USA). P-selectin expression was determined by using PE-conjugated CD62 and CD42a-FITC antibodies. Convulxin activated or non activated platelets of dasatinib/nilotinib treated CML patients were fixed by paraformaldehyde and stained by above mentioned monoclonal antibodies.

### 4.5. Thrombin Generation Assay

Thrombin generation was measured in PRP using Fluoroskan Ascent FL fluorimeter with Thrombinoscope reagents and software (Thrombinoscope BV, Maastricht, The Netherlands). Assays were carried out according to the manufacturer’s instructions.

Into wells of a black plate, 80 µL of pretreated PRP and 20 µL of standard preparation containing 1 pM recombinant tissue factor (PRP reagent)/ Thrombin calibrator were pipetted, and after incubation for 10 min at 37 °C, the thrombin generation was started by adding 20 µL of FluCa (fluorogenic substrate and calcium in buffer). Fluorescence was detected and the thrombin generation curve was generated. The kinetics of thrombin generation was characterized by lagtime, time to peak, while the quantity of generated thrombin was described by thrombin peak end endogenous thrombin potential (ETP).

### 4.6. Clot Retraction

In the in vitro experiments, 900 μL of PRP was preincubated with 100 μL of buffer control or different concentrations of dasatinib or nilotinib for 10 min at 37 °C in a water bath and subsequently was activated by convulxin for 15 min at 37 °C. In a glass tube, 1000 μL of TKI-pretreated and activated PRPs from an in vitro experiment, and similarly to this, 1000 μL PRPs from dasatinib/nilotinib treated CML patients were incubated with 100 μL of 250 mM CaCl_2_ (at a final concentration of 22.7 mM) for 60 min at 37 °C in a water bath. At the end of incubation, photos were taken to document clot formation and the volume of the extruded serum was determined.

### 4.7. Western Blot Analysis

Platelets of dasatinib or nilotinib treated CML patients were purified by gel filtration [17] that was performed on Sepharose CL-2B column. In the in vitro experiments, control platelets were first gel filtrated and then were pretreated by dasatinib/nilotinib and subsequently activated by convulxin.

Gel filtrated platelets (4 × 10^7^ from each sample) were lysed with lysis buffer containing 1% TritonX-100 in PBS supplemented with a cocktail of tyrosine phosphatase inhibitor from Sigma (St. Louis, MO, USA). Platelet lysates were separated by polyacrylamide gel electrophoresis and subjected to western blotting. Specific phosphorylation of Lyn, Fyn, and Src kinases were visualized, using phosphospecific antibodies (Ab) and biotinylated secondary Ab followed by avidin-biotin complex for 30 min. Bands were demonstrated by enhanced chemiluminescence (ECL).

### 4.8. Statistical Analysis

GraphPad Prism version 4.0 program was used for the statistical analysis. Data distribution was evaluated by Kolmogorov–Smirnov test. The statistical significance of the differences between groups of in vitro experiment was analyzed by one-way ANOVA in case of Gaussian distribution, and by Kruskal–Wallis test in case of non-Gaussian distribution, as appropriate. The statistical significance of the differences between before (0 h) and 1 h after drug administration groups of CML patients was analyzed by paired Student’s t-test in case of Gaussian distribution and by Wilcoxon rank test in case of non-Gaussian distribution. Differences were considered significant when p values were below 0.05.

## Supplementary Materials

Supplementary materials can be found at https://www.mdpi.com/1422-0067/20/21/5430/s1.
