# Supplementary material for: Dasatinib Inhibits Procoagulant and Clot Retracting Activities of Human Platelets
Source: Int J Mol Sci. 2019 Oct 31;20(21):5430. doi: 10.3390/ijms20215430 (PMC6862041; doi:10.3390/ijms20215430)
Supplement: Supplementary file 1 [file ijms-20-05430-s001.pdf]

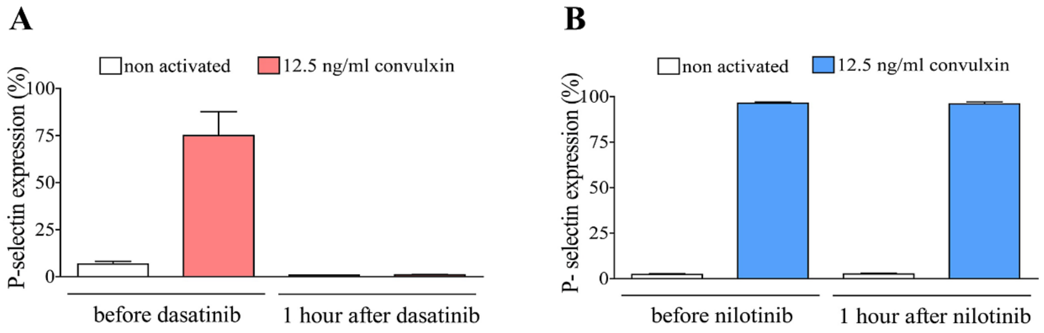

Figure S1. Dasatinib completely abolished the effect of convulxin on alphagranule excretion.Samples were from dasatinib (n=3) or nilotinib (n=3) treated CML patients before and at 1 h after witnessed drug administration. Platelets of patients were stimulated with convulxin in PRP. Alphagranule excretion was investigated by monoclonal antibody againts P-selectin. Columns show the mean and SEM.
